# The anti-proliferative effect of apricot and peach kernel extracts on human colon cancer cells in vitro

**DOI:** 10.1186/s12906-019-2437-4

**Published:** 2019-01-29

**Authors:** Wagheda Cassiem, Maryna de Kock

**Affiliations:** 1 School of Natural Medicine, University of the Western Cape, Robert Sobukwe road, PO Box x17, Cape Town, Bellville, 7535 South Africa; 2 Department of Medical Biosciences, University of the Western Cape, Robert Sobukwe road, PO Box x17, Cape Town, Bellville, 7535 South Africa

**Keywords:** Apricot kernel, Peach kernel, Amygdalin, Colon cancer, S-phase block

## Abstract

**Background:**

Colorectal malignant neoplasms is one of the leading causes of death in both men and women in the developed world and the incidence has recently increased markedly in South Africa. Studies have highlighted the beneficial effects of Amygdalin, a cyanogenic compound found in both peach and apricot kernels, in its ability to suppress the development of colon cancer. The focus of this study was to investigate the potential anti-proliferative properties of various apricot and peach kernels extractions from South Africa and China and to monitor alterations in cell cycle kinetics in colon cancer cells.

**Methods:**

Studies were conducted on HT-29 colon cancer cells. The interactive role of three different kernel extractions on the modulation of cell proliferation, apoptosis and cell cycle progression was monitored over 24, 48 and 72 h periods.

**Results:**

After 24 h, all extracts of the South African apricot kernels had a dose related bi-phasic proliferative effect on the HT-29 cells. It stimulated cell proliferation at the lowest and highest concentrations while at 500 μg/mL it inhibited cell proliferation. In contrast, after 72 h, the low concentration inhibited cell proliferation while the 500 μg/mL extracts stimulated cell proliferation. Morphological changes were observed in cells incubated with Chinese kernel extracts after 24 h and South African kernel treatment (1000 μg/mL) after 72 h. A possible intra-S-phase block after 24 and 48 h exposure to South African hydrophilic kernel extracts was observed. This transient block that is more concerned with tolerating and accommodating damage during replication rather than repairing it, could explain the initial anti-proliferative effects observed after 24 h exposure to the various Chinese kernel extract concentrations.

**Conclusion:**

Abrogation of the block by exhaustion of the cyanide production, most likely allowed the cells to resume the cell cycle and continue into mitosis, whereas low ATP levels caused by the presence of amygdalin in the kernels, can also cause the induction of pycnosis or necrosis. These results highlight the possible mechanisms of growth inhibition by amygdalin containing extracts and may contribute towards the development of dietary anti-cancer therapies.

## Background

The National Cancer Registry of South Africa has recorded that colorectal cancer (CRC) is one of the top ten most common cancers in both men and women [1]. Although advances in radiotherapy, chemotherapy and surgery has improved survival rates, approximately 1 in 17 people over the age of 50 years will develop colon cancer in their lifetime, with more than 50% of these individuals eventually dying from the disease [1]. Colon cancer is nutrition dependent [2] with alcohol consumption accounting for the greatest risk factor [3, 4], followed by a high-fat diet, poor dietary fibre intake, obesity, smoking and lack of exercise [2]. Colon cancer may be treated by the resection of the colon, chemotherapy, targeted therapy, hormonal therapy and immunotherapy [5], with pharmaceutical hormonal drugs resulting in undesirable adverse effects [6]. The efficacy of 5- Fluorouracil (5-FU), the mainstay of colon cancer treatment [7] do not have a consistent drug response in patients [8].

Ruan et al. (2006) [9] have shown that apricot and peach kernels dispensed in its raw form, induce apoptosis and differentiation of tumour cells. Human DU145 and LNCaP prostate cancer cells showed morphological characteristics associated with apoptosis as well as increased Bax expression and caspase-3 enzyme activity after treatment with apricot kernel extracts [9]. The apricot and peach kernels are composed of glycosides including amygdalin, fixed oils including oleic acid and linoleic acid, volatile oils such as benzaldehyde [﻿10﻿]. In addition, the peach and apricot kernel has polyphenols such as flavanoids [10﻿] and gallic acid respectively [11]. Epidemiological studies support evidence that colon cancer is preventable by adjusting the diet [12] and a protective effect is attributable to polyphenols and foods such as fruits and vegetables [﻿13﻿]. Wu et al. (2011) had found that high content of phenolic compounds (4.1593 mg GAE/g) contribute to the antioxidant activity of the kernels oil [﻿14﻿]. The presence of oleic and linoleic acids in the peach kernel enhances its nutritional and medicinal value [15]. Both amygdalin and its patented form, Laetrile®, have been promoted and sold as “vitamin B-17”, although neither compound is a vitamin.

Cyanogenic glycosides are considered non-toxic until cyanide is released. This usually occurs as a result of enzymatic hydrolysis by β-glucosidases following grinding of plant tissue which activates intracellular β-glucosidases, or by the gut micro-flora. This reaction can also result from chewing. Amygdalin is a cyanogenic glycoside plant compound found in kernels of the Rosacea family (Persica Semen and Armeniacea Semen) that release cyanide upon enzymatic metabolism. Cyanide acts through the inhibition of cytochrome-c oxidase in the respiratory electron transport chain of the mitochondria, impairing both oxidative metabolism and the associated process of oxidative phosphorylation, thereby causing death through energy deprivation and oxygen uptake [16]. The rationale for using amygdalin / laetrile rests on the fact that non-malignant cells have higher levels of a liver mitochondrial enzyme, rhodanese, a sulphur transferase, which catalyzes the detoxification of cyanide by sulphuration in a double displacement mechanistic reaction [﻿17﻿]. In animal tissue rhodanese is multifunctional, but the physiological role is to catalyze the formation of thiocyanate that is excreted by the kidneys [1﻿8﻿]. The apricot and peach kernel’s role in nutrition and as a substance with medicinal properties makes it a target for anti-cancer drugs.

In South Africa, practitioners of Traditional Chinese Medicine prescribe apricot and peach kernels imported from the China as part of herbal formulations to their patients. It would therefore be of value to both the practitioner and the patient to know whether the kernels from South Africa could have similar effects.

The aim of this research was to investigate the potential anti-proliferative properties and possible cytotoxic effects of apricot and peach kernel extracts from South Africa and China on HT-29 colon cancer cells. The study included the evaluation of cell cycle progression after exposure to the various kernel extracts.

## Methods

### Cell culture

HT-29 colon cancer cells (ATTC®HTB-38™, Middlesex TW11 OLY, United Kingdom), a gift from Prof WCA Gelderblom (CPUT, Bellville, Cape Town), were cultured in Dulbecco’s Minimum Essential Medium F12 (Thermo Fisher Scientific, South Africa) supplemented with 15% heat inactivated Fetal Bovine Serum (FBS) (GIBCO), penicillin (100 μg/mL) and streptomycin (100 μg/mL) (GIBCO). The cells were grown and maintained in sterile 25cm^2^ tissue culture flasks at a 5% CO_2_, 95% humidified air atmosphere at 37 °C. Tissue culture flasks, serological pipettes and filters were obtained from BIOCOM/Biotech.

### Kernel extracts preparation

Raw Chinese apricot and peach kernels were obtained from China through a private Traditional Chinese Medicine (TCM) practitioner, Dr. Li Chunlan, Newlands, Cape Town, South Africa. The South African kernels were obtained from De White Industries (Pty) Ltd. (P.O Box 400, Montagu, Western Cape, South Africa) producers and exporters. All kernels were grounded using a household coffee blender. The Chinese apricot (CAK), Chinese peach (CPK), South African apricot (SAK) and South African peach (SPK) organic extractions, namely total (tot), lipophilic (lipo) and hydrophilic (hydro) extractions, were obtained using 80% ethanol. Organic extractions were achieved by evaporating ethanol extracted kernels using a desktop rotatovapour (Buchi rotavapor R-114) at 55 °C to obtain a viscous oily liquid and stored at − 20 °C. To make up a stock solution of the respective CAK, CPK, SAK and SPK kernel extractions, 1 g of the respective oils were added to 9 mL of DMSO to make up a final volume of 10 mL.

Dimethyl-Sulfoxide (DMSO) (9 mL) and 1 g of the respective oils was used to constitute the respective concentrations of 100, 500 and 1000 μg/mL for the organic extractions. The kernel extractions hereafter are referred to as the treatment. Routinely the vehicle concentration (Ethanol/DMSO) was limited to 0,1% in the low concentration of extracts used and 0,3% in the high concentrations of extracts used in the experiments.

### Cell proliferation

HT-29 cells were seeded in 96 well plates at 5000 cells/well and incubated for 24 h (h). The cells were exposed to the organic extracts at concentrations of 100, 500 and 1000 μg/mL respectively for 24 h, 48 h and 72 h. A crystal violet assay was used to determine cell proliferation [19] and the absorbance of the samples was analysed at 570 nm using a Glomax plate reader.

### Morphological studies

#### Light microscopy: Haematoxylin and eosin staining

Haematoxylin and Eosin cell staining (H and E) was conducted as a standard microscopic technique for qualitative evaluation of cellular morphology. Equal numbers of cells were seeded on sterilized coverslips in 6-well culture plates and left to attach for 24 h. Thereafter the cells were exposed to the various concentrations of the organic extracts for 24 h, 48 h and 72 h respectively. The coverslips with attached cells were removed and the cells were fixed in Bouin’s solution and stained with Haematoxylin and Eosin using a standard procedure [20]. The cells were viewed with a Nikon Eclipse 50i light microscope. Possible cytotoxic effects of the kernel extracts, including the presence of apoptosis, were studied.

#### Fluorescent microscopy: Apoptosis detection

Using fluorescence, possible cell death including apoptosis can be observed. Cells were seeded on sterilized coverslips placed in 6-well culture plates. After incubation for 24h to allow attachment, the cells were treated with the specific organic extractions (500 μg/mL) for 24h, 48h and 72h. After 24h and 48h respectively the cells attached to the cover slips were stained with 1 μg/mL Hoechst 33342 (Sigma) in medium for 30 min at 37C^o^. Following this, the cells were washed three times with PBS and mounted using a solution of 0.5% p-phenylenediamine in 20mM Tris (pH 8.8) and 90% glycerol. The cells were analysed by a Nikon fluorescent microscope using a 450nm emission filter.

#### Flow cytometry: Cell cycle progression

Cell cycles G1, S, G2 were analysed in HT 29 cells using propidium iodide to stain the nucleus in order to determine the amount of DNA. Cells were seeded in 25cm^2^ flasks and incubated at 37 °C for 24 h after which the cells were treated with 100, 500 and 1000 μg/mL extracts for 24 h, 48 h and 72 h. Cells were harvested and pelleted by centrifugation at 3500 rpm/6 min. The supernatant poured off and the 3 mL of ice cold 95% ethanol was added to each tube in a drop wise manner whilst vortexing each tube till the cell-ethanol mixture was homogenous. The specimens were stored at − 20 °C overnight. The ethanol was removed by centrifugation at 3500 rpm/6 min and the cells washed with 1 mL PBS at 6 min intervals. The sediment was re-suspended in 1 mL hypotonic DNA staining buffer, Propidium Iodide (PI) / RNase [Roche], and stored at 4 °C for 30 min before reading the specimens using a Becton Dickinson FACS Calibre benchtop flow cytometer with a 488 nm Coherent laser. For each sample at least 10,000 events were collected and aggregated cells were gated out. The number of gated cells in the G_1,_ G_2_/M and S-phase is represented as %. (RNase solution: 250 mL distilled water, 0.25 g Tri NaCitrate, 750 μL Triton X-100, 0.025 g PI and 0.005 g RNase A).

### Statistics

Statistical analysis of the quantitative data was done by GraphPad Prism (GraphPad Prism version 6.04 for Windows, GraphPad Software, San Diego California USA, www.graphpad.com). Cell viability studies were repeated thrice, with sample size (n) — 12 in each experiment and analyzed by means of two-way ANOVA followed by Tukey’s multiple comparisons test. A *P*-value of less than 0.05 was accepted as significant. Means of quantitative experiments are presented in bar charts, with T-bars referring to the standard error of the mean (SEM). All experiments included a set of appropriate controls. Changes in cellular kinetics and morphology were carried out to illustrate possible explanations for the changes in cellular proliferation and not statistically analysed. The software used for acquisition of cell cycle progression data, was Cellquest Pro version 5.2.1. Analysis and results obtained of acquired data was done with Modfit version 3.3 software and not presented as mean ± SD.

## Results

### Cell proliferation

**Fig. 1:**
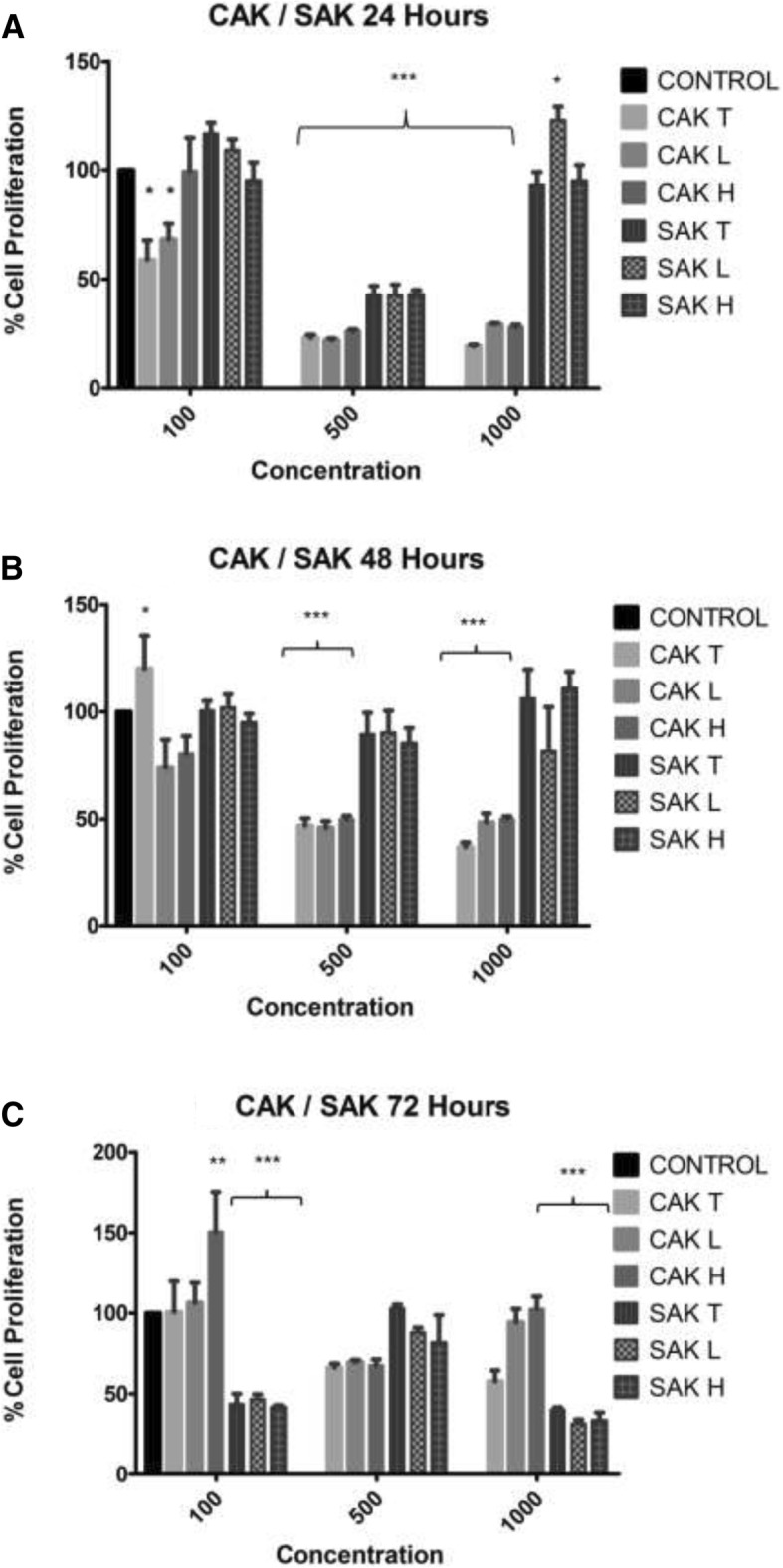
Percentage of HT-29 cells as determined by crystal violet assay in 96-well plates following 24 (**a**), 48 (**b**), and 72-h (**c**) exposure to CAK and SAK extracts. Data is shown as mean ± SEM of three separate experiments. Significant differences between treated and control samples are indicated by *P ≤ 0.05, ** *P* ≤ 0.01, *** *P* ≤ 0.001 compared to respective controls (Two-way ANOVA followed by Tukey’s multiple comparisons test)

Cell numbers of the HT-29 colon cancer cells were affected differently by the kernels of different origin. Cell numbers decreased in a time and dose-dependent manner when exposed to the Chinese apricot kernel extracts for 24 h and 48 h (Fig. 1a and b). After 72 h however, CAK-hydro (100 μg/mL) and CAK-hydro and CAK-lipo (1000 μg/mL) stimulated cell proliferation. All the extracts of the South African apricot kernels (SAK) had a dose related bi-phasic proliferative effect on the HT-29 cells (Fig. 1). The lowest and highest concentrations of the SAK extracts had an outspoken stimulatory effect on cell proliferation after 24 h while at 500 μg/mL, it significantly inhibited cell proliferation (Fig. 1a). In contrast, after 72 h, the lowest and highest concentrations (100 & 1000 μg/mL) of all SAK extracts significantly (*p* ≤ 0.001) inhibited cell proliferation while the 500 μg/mL extracts now stimulated (*p* ≤ 0.001) cell proliferation (Fig. 1c). For the 48 h exposure period, the SAK extracts followed the same trend as observed in the 24 h exposure samples, however the effect was not significant (Fig. 1b).

**Fig. 2:**
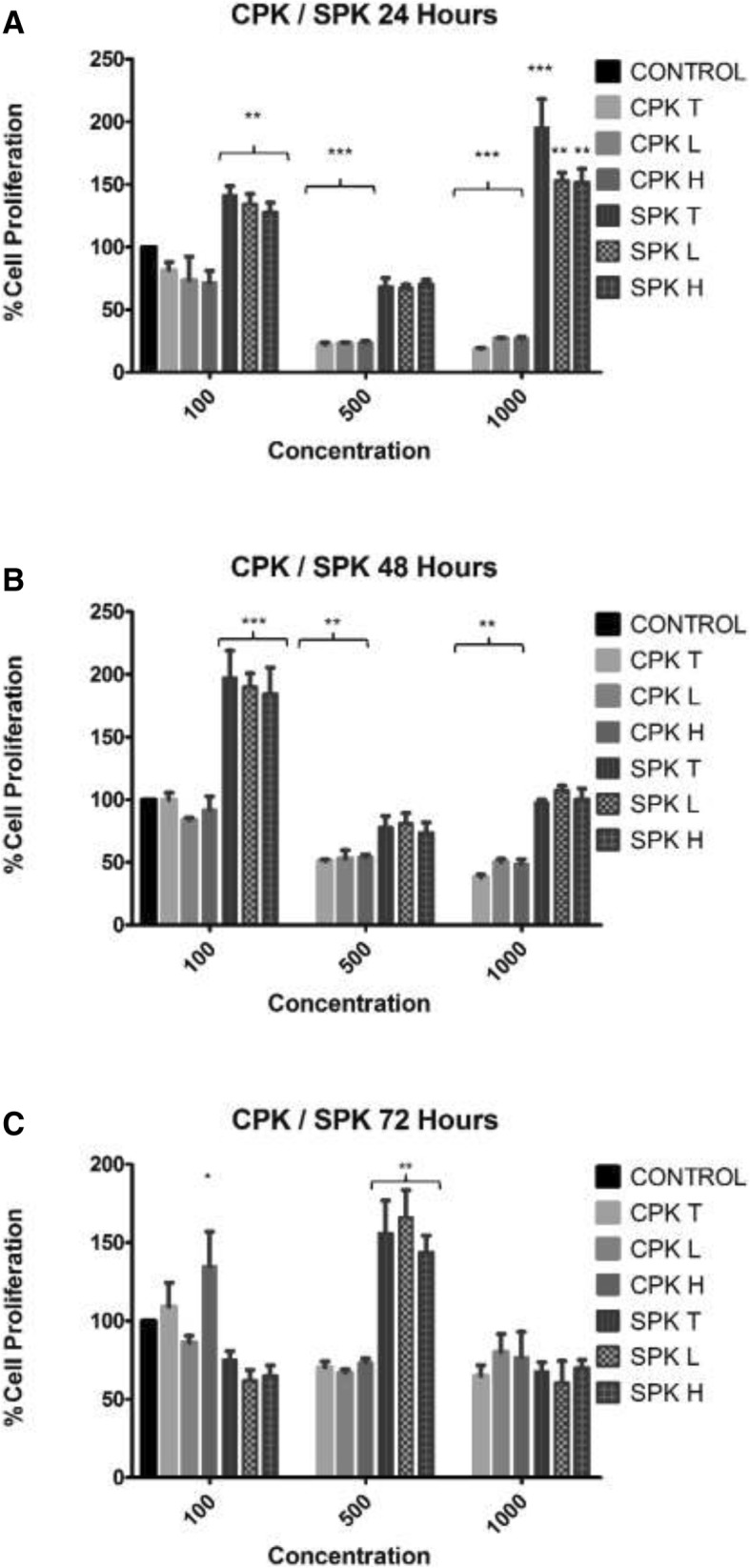
Percentage of HT-29 cells as determined by crystal violet assay in 96-well plates following 24 (**a**), 48 (**b**), and 72-h (**c**) exposure to CAK and SAK extracts. Data is shown as mean ± SEM of three separate experiments. Significant differences between treated and control samples are indicated by *P ≤ 0.05, ** *P* ≤ 0.01, *** *P* ≤ 0.001 compared to respective controls (Two-way ANOVA followed by Tukey’s multiple comparisons test)

For the peach kernels (Fig. 2), the South African kernel extracts (100 μg/mL) at 24 and 48 h, the 1000 μg/mL (24 h) and the 500 μg/mL (72 h) induced an outspoken increase in cell proliferation. These results are similar to that observed with the South African apricot kernel extracts. The most outspoken inhibition of cell proliferation by the CPK organic extracts was induced after 24 h and 48 h by the 500 and 1000 μg/mL concentrations (Fig. 2a and b). The inhibitory effects induced by the total and lipophilic extracts is possibly due to the presence of glycosides, fixed and volatile oils as well as the high nutritional value due to the high lipophilic extract percentage.

In contrast to the inhibitory effect seen after 24 h and 48 h, the cells exposed to CPK-H after 72 h showed an increase in cell proliferation (Fig. 2c).

### Morphological effects

**Fig. 3:**
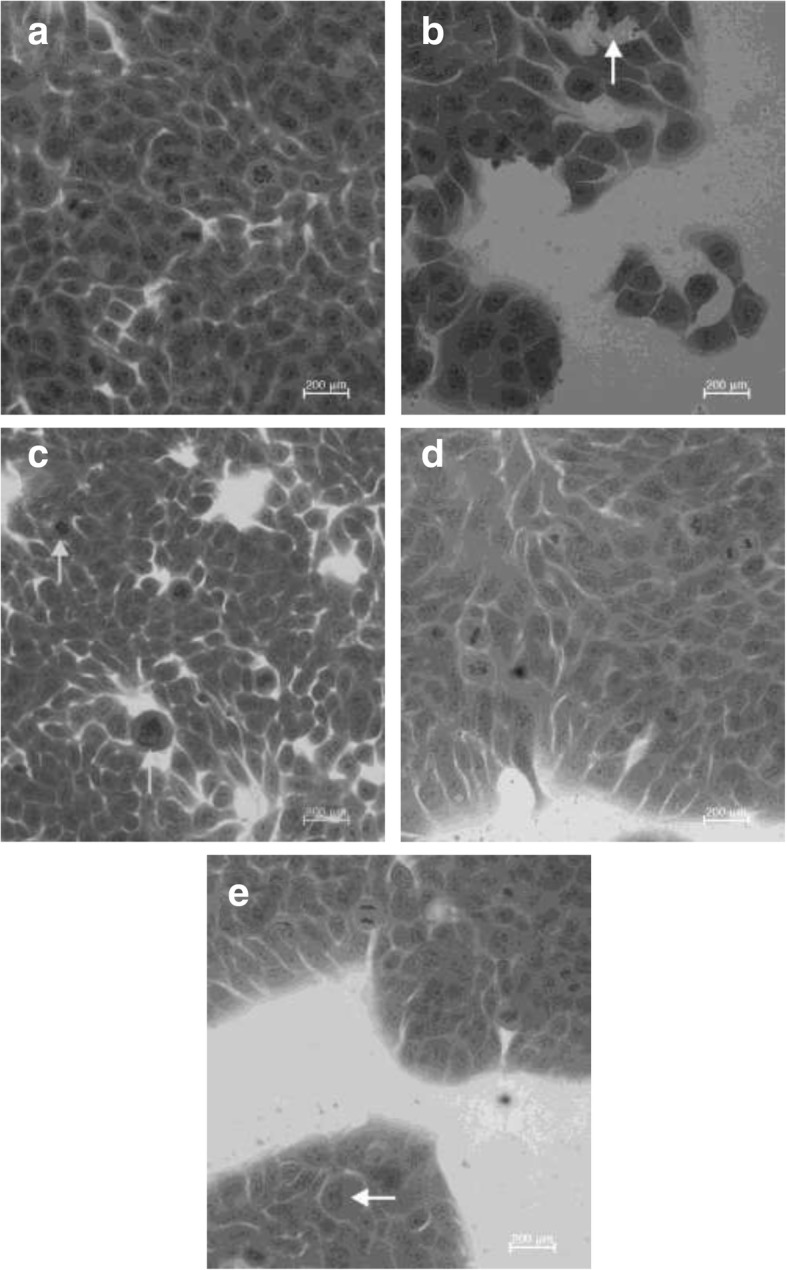
Untreated cells (**a**) and HT-29 cells exposed to 100 μg/mL CPK-H (24 h) (**b**) and CAK-Tot extract (48 h) (**c**), 1000 μg/mL SPK-Tot (**d**) and 500 μg/mL SAK-H (72 h) extracts (**e**). HT-29 colon cancer cells, mostly found in interphase, form colonies of round to elliptical shapes. Round nuclei with 2–4 prominent nucleoli with uniform sized cells are visible. Treated HT-29 colon cancer cells (**b**) show membrane blebbing (arrows) indicative of apoptosis formation, hypercondensed chromatin (**c**) and cellular shrinkage (**d** and **e**). Figure 3a, c, d, and e (magnification 40x), and 3b (magnification 100x)

Colony forming HT-29 colon cancer cells show characteristic round to elliptical shapes (Fig. 3a). In response to the treatment with the 100 μg/mL CPK-H (24 h) (Fig. 3b) cells displayed cell membrane blebbing (arrow) possibly due to changes in membrane permeability; to 100 μg/mL CAK-T extract (48 h) (Fig. 3c) irregularly shaped cells and 1000 μg/mL SPK-Tot and 500 μg/mL SAK-H (72 h) (Fig. 3d and e) cellular shrinking. These changes could possibly be associated with cells undergoing apoptosis. After 72 h however, the cells seem to resume their initial growth appearance and increase in colony size.

**Fig. 4:**
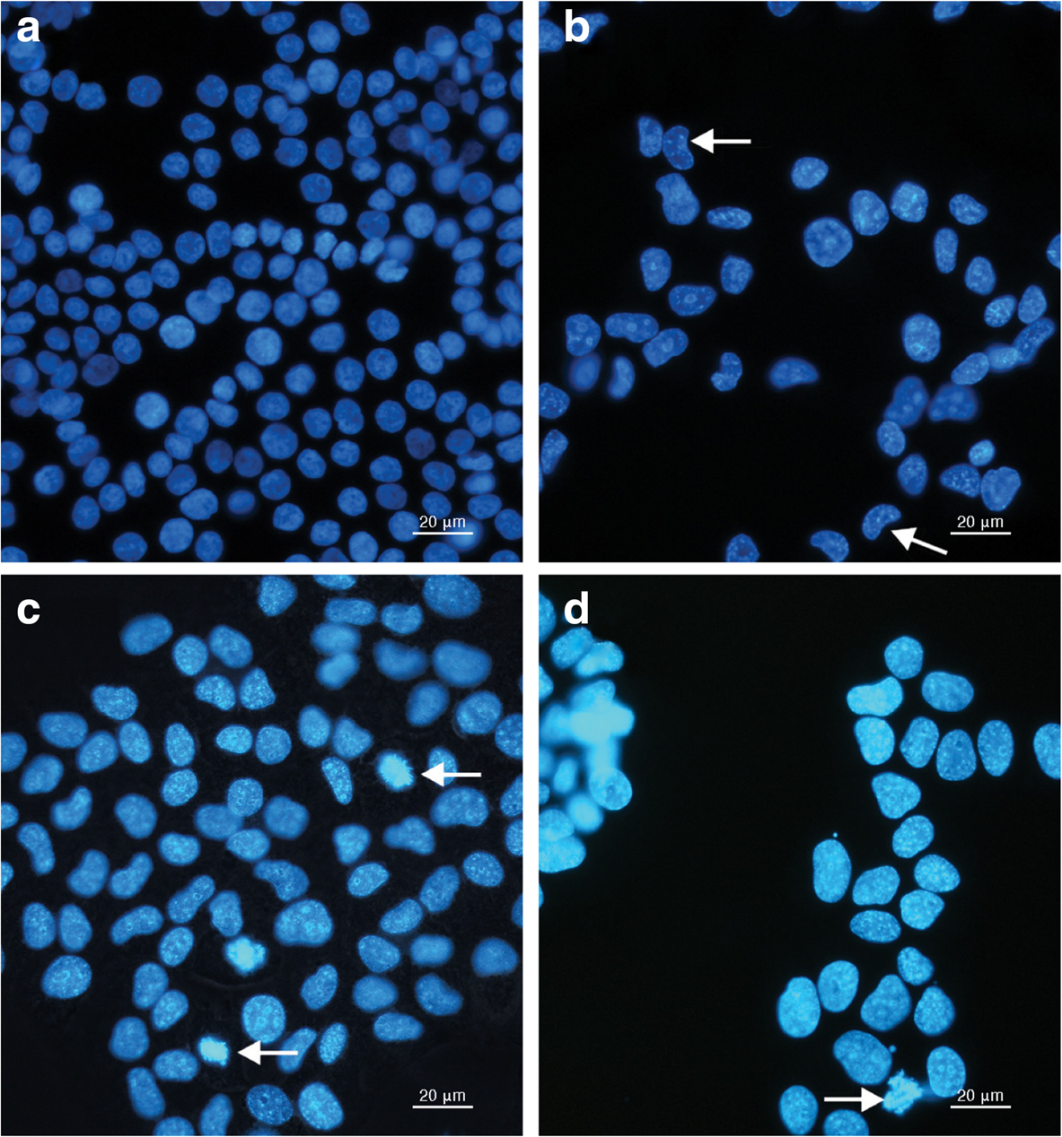
Control HT 29 cells (**a**), cells treated with 500 μg/mL SPK-H (48 h) (**b**), 500 μg/mL SAK-H (48 h) (**c**) and 500 μg/mL SPK-L (72 h) (**d**) and stained with Hoechst (magnification 40x). All pictures are typical of three independent experiments each performed under identical conditions

To verify the possible presence of apoptosis, treated cells were stained with Hoechst33342. Only a few apoptotic cells were observed, negating the hypothesis that the decrease in cell numbers in the SAK and SPK treated cells was due to apoptosis induction (Fig. 4a–d).

### Cell cycle kinetics

Compared to the control in Table 1 the SPK extracts showed a consistent increase in the number of cells in the S-phase. After 24 h exposure, the SAK hydrophilic extract at 100 μg/mL (S = 86.4%), and 65.57% (500 μg/mL) and 74.47% (1000 μg/mL) showed the most significant accumulation of cells in the S-phase (Table 1). Similar increases in the S-phase cell fraction after SAK and SPK kernel treatments were observed after the 48 h exposure period with the largest build-ups caused by SPK-H (100 μg/mL) at 76.08%, 500 μg/mL SAK-L at 70.74% and 1000 μg/mL SPK-T at 77.62% (Table 2). Cells treated with the CPK organic fractions resulted in an increase in the number of cells in the S-phase (S = 56.73%) when exposed to 500 μg/mL CPK organic extractions after 72 h exposure (Table 3). The percentage values of SAK and SPK extract exposed cells in the G_1_ and S-phases vary greatly between each other indicating a higher variance as compared to the CAK and CPK exposed cells.

**Table 1:** The percentage distribution of the control and CAK, CPK, SAK and SPK treated HT-29 cells after 24 h. The SAK and SPK extracts caused an outspoken increase in the percentage of cells that arrested in the S-phase of the cell cycle indicating a higher mean ± SD as compared to the CAK and CPK exposed cells. CPK extracts induced its highest percentage of cells in the S-phase at a concentration of 1000 μg/mL

Time and dosage	Kernel sample	Cell cycle phases		
		G1	S	G2
24h100μg/mL	Control	85.2	13.88	0.91
	CAK T	90.09	7.99	1.92
	CAK L	94.8	3.76	1.44
	CAK H	90.64	7.81	1.55
	CPK T	75.68	**24.32**	0
	CPK L	80.17	18.41	1.42
	CPK H	82.36	16.12	1.52
	SAK T	62.08	**37.92**	0
	SAK L	62.13	**38.87**	0
	SAK H	13.6	**86.4**	0
	SPK T	35.53	**64.47**	0
	SPK L	33.2	**66.8**	0
	SPK H	43.75	**56.25**	0
24h500μg/mL	CAK T	88.78	9.83	1.4
	CAK L	84.3	14.43	1.27
	CAK H	87.6	10.45	1.94
	CPK T	87.69	10.31	2
	CPK L	86.9	10.67	2.43
	CPK H	86.69	11.98	1.33
	SAK T	39.87	**60.13**	0
	SAK L	79.13	**20.87**	0
	SAK H	34.43	**65.57**	0
	SPK T	65.75	**34.25**	0
	SPK L	78.12	**21.88**	0
	SPK H	49.02	**50.98**	0
24h1000μg/mL	CAK T	78.47	17.38	4.15
	CAK L	78.51	15.88	5.61
	CAK H	81.06	16.95	1.99
	CPK T	57.11	**38.17**	4.72
	CPK L	70.1	**29.9**	0
	CPK H	50.67	**42.67**	6.65
	SAK T	39.81	**60.19**	0
	SAK L	84.43	15.57	0
	SAK H	25.53	**74.47**	0
	SPK T	89.71	10.29	0
	SPK L	42.88	**57.12**	0
	SPK H	50.33	**49.67**	0

numbers in bold indicate % values almost double and more than the Controls values for cells in the S-phase

**Table 2:** The percentage distribution of the control and CAK, CPK, SAK and SPK treated HT-29 cells after 48 h. The SAK and SPK extracts caused an outspoken increase in the percentage of cells that arrested in the S-phase of the cell cycle indicating a higher mean ± SD as compared to the CAK and CPK exposed cells

Time and dosage	Kernel sample	Cell cycle phases		
		G1	S	G2
48h100μg/mL	Control	8.,47	15.3	1.24
	CAK T	71.05	**28.95**	0
	CAK L	89.91	10.09	0
	CAK H	86.82	12.63	0.55
	CPK T	87.31	12.02	0.67
	CPK L	77.04	20.71	2.25
	CPK H	69.05	**29.12**	1.83
	SAK T	51.56	**45.32**	3.12
	SAK L	84.06	13.12	2.83
	SAK H	81.57	16.33	2.1
	SPK T	51.39	**48.61**	0
	SPK L	47.06	**52.94**	0
	SPK H	23.92	**76.08**	0
48h500μg/mL	CAK T	81.41	17.74	0.85
	CAK L	85.68	12.53	1.79
	CAK H	83.12	16.33	0.55
	CPK T	8725	11.34	1.4
	CPK L	87.88	9.56	2.56
	CPK H	86.21	10.82	2.97
	SAK T	77.9	16.71	5.39
	SAK L	29.26	**70.74**	0
	SAK H	66.34	**33.66**	0
	SPK T	81.08	15.06	3.86
	SPK L	71.66	**28.34**	0
	SPK H	58.12	**41.88**	0
48h1000μg/mL	CAK T	81.14	15.16	3.71
	CAK L	86.3	11.78	1.92
	CAK H	83.58	13.66	2.75
	CPK T	87.65	11.41	0.94
	CPK L	86.93	12.3	0.77
	CPK H	86.92	13.08	0
	SAK T	54.87	**45.13**	0
	SAK L	62.13	**37.87**	0
	SAK H	58.27	**41.73**	0
	SPK T	22.38	**77.62**	0
	SPK L	62.89	**37.11**	0
	SPK H	53.46	**46.54**	0

numbers in bold indicate % values almost double and more than the Controls values for cells in the S-phase

**Table 3:** The percentage distribution of the control and CAK, CPK, SAK and SPK treated HT-29 cells after 72 h. Although the SAK and SPK extracts induced an outspoken increase in percentage of cells that arrested in the S-phase, for most of the extractions the effect was overcome

Time and dosage	Kernel sample	Cell cycle phases		
		G1	S	G2
72h100μg/mL	Control	83.51	15.61	0.88
	CAK T	81.88	15.92	2.2
	CAK L	77.11	21.72	1.17
	CAK H	80.06	17.43	2.51
	CPK T	79.07	19.34	1.59
	CPK L	80.85	18.43	0.72
	CPK H	86.88	11.81	1.31
	SAK T	83.19	16.04	0.76
	SAK L	70.39	**29.61**	0
	SAK H	77	23	0
	SPK T	89.78	10.22	0
	SPK L	36.56	**63.44**	0
	SPK H	79.39	20.61	0
72h500μg/mL	CAK T	81.14	15.16	3.71
	CAK L	86.3	11.78	1.92
	CAK H	83.58	13.66	2.75
	CPK T	86.09	10.99	2.92
	CPK L	85.08	12.34	2.57
	CPK H	43.27	**56.73**	0
	SAK T	89.14	10.86	0
	SAK L	85.23	14.77	0
	SAK H	79.97	17.73	2.29
	SPK T	83.64	16.36	0
	SPK L	62.86	**37.14**	0
	SPK H	84.42	15.58	0
72h1000μg/mL	CAK T	86,43	11.13	2.45
	CAK L	86.39	10.57	3.04
	CAK H	87.47	8.86	3.67
	CPK T	82.31	16	1.69
	CPK L	88.12	9.49	2.39
	CPK H	87.31	9.24	3.45
	SAK T	74.9	**24.54**	0.56
	SAK L	82.83	14.9	2.27
	SAK H	86.51	11.97	1.53
	SPK T	66.84	**33.16**	0
	SPK L	89.59	10.41	0
	SPK H	96.66	3.23	0.11

numbers in bold indicate % values almost double and more than the Controls values for cells in the S-phase

## Discussion

Colon cancer treatment entails surgical removal of the tumor that is followed by chemotherapy with or without radiation. However, the notorious effect of such treatments has in many cases exacerbated the overall diminishing health and quality of life of the recipients. Although early detection has improved survival, the chemotherapeutic agents used in the treatment of colon cancer have not proven to be efficient in all patients. Studies strongly correlate the relationship between dietary constituents and the risk of specific cancers [10, 13, 14]. With colon cancer, a relation commonly found in epidemiological studies is an increase in risk associated with a high fat and low fibre dietary pattern [﻿14﻿]. Certain dietary fatty acids (FA) can irritate the colon by increasing pro-inflammatory responses. The presence of glycosides such as amygdalin, oleic and linoleic acid in the kernels render them as possible chemotherapeutic agents. Replicating cancer cells have an increased requirement for lipids for membrane formation, hence dietary interventions with fatty acids possessing anti-carcinogenic properties represent a novel, practical and relatively safe approach to reduce proliferation of colorectal cancer [21]. Inhibition of cell proliferation and the induction of apoptosis could be an important approach in chemotherapeutic treatment. Understanding how dietary components regulate proliferation and cell survival could play a critical role in development of new agents that can prevent and treat cancer with reduced risk of toxicity. Conventional chemotherapy lacks aqueous solubility, selectivity and due to its nonspecific targeting of cancer cells, damages rapidly proliferating normal cells [6]. Epidemiological studies have revealed that colon cancer is preventable by adjusting the diet leading to a reduction in the incidence. Thus identification of chemopreventive compounds in commonly consumed natural dietary sources such as fruits, vegetables and teas is important in that it provides a means of everyday cancer prevention. Laboratory studies have indicated a positive association between cancer reduction and intake of dietary plant foods which is attributable to the presence of phytochemicals [22]. One group of phytochemicals that has shown potential in chemoprevention studies are plant polyphenols [23].

Plant glycosides, which are flavonoids and polyphenolic, have a bio-action, are larger and chemically more complex than in mammals, are therapeutic in small doses but toxic in larger doses [﻿24﻿]. They thus have a narrower therapeutic index. This is reflected in the time-dose-dependent manner as well as the type of extract preparation and its effects on the HT-29 colon cancer exposed cells. Reflecting the anti-proliferative effect at low doses at 24 h and 48 h exposure and a recovery after 72 h exposure to the kernel extractions.

This investigation demonstrated that organic extracts of apricot and peach kernels of Chinese and South African origin may significantly reduce cell viability, inhibit cell proliferation, induce morphological changes and affect cell cycle progression of the HT-29 human colon cancer cell line. These findings agree with reports showing growth inhibiting, anti-proliferative and pro-apoptotic effects of amygdalin on promyelocytic leukaemia [25], colon cancer [26], cervical cancer [﻿27﻿], and bladder cancer cells [28]. As opposed to previous research where the isolated cyanogenic compound was used, in the present research the whole kernel was used. This may have contributed to the slight variation in the results. The rationale for using the whole kernel was based on the principle that the whole is better than the parts, whose constituents act synergistically as a whole. The method of preparation (in the form of an herbal medicine as administered by a Chinese medicine practitioner) and consumed as a food supplement were other factors considered. Since the Chinese apricot and peach kernels were bought from a Chinese medicine practitioner it was expected that there would be less fixed and volatile oils as a result of the preparation process of the kernels. The aim of the present study was to examine the influence of CAK/SAK as well as CPK/SPK on colon cancer cell proliferation to determine whether these compounds affects cell morphology and cellular kinetics, thus mechanisms influencing cell growth.

Although the organic extracts of both the apricot and peach kernels affected cell proliferation, it was the Chinese apricot and peach total and lipophilic kernel extracts that had the most outspoken inhibition on cell viability after 24 h. This may be due to the specific ratio of amygdalin and the presence of volatile oils, fixed oils and glycosides in the kernels originating from China. The glycosides in the kernels causes polarisation of water insoluble compounds that could possibly also contribute to the growth inhibitory effect [﻿24﻿]. The antioxidant potential of polyphenols depends on the chemical structure particularly the positions of the hydroxyl groups [29]. Thus the fixed and volatile oils may be greatly reduced in hydrophilic extracts possibly resulting in a reduced inhibitory effect by the hydrophilic extracts compared to the total and lipophilic extracts. The HT-29 cancer cell growth inhibitory effect of the CAK and CPK treatments was abrogated after 72 h exposure with an increase in cell proliferation observed in most of the treated samples. In contrast with the anti-proliferative effects observed after treatment with CAK and CPK, the South African kernel extracts stimulated cell proliferation in the 24 and 48 h exposure periods. The bi-phasic stimulation/inhibition by these extracts (Figs. 1 and 2a, b) would certainly impact on the use of these kernels in patients as the dose effect could potentially affect the treatment. The aggressive stimulation by low and high doses SAK/SPK (24 h) (Figs. 1a and 2a) in contrast to the absence of the stimulatory effects of the 500 μg/mL can possibly be related to the biphasic nature of the respective components in these kernels. Several forms of cell death may typically be induced within the same tissue although apoptosis is the fastest, while other forms, like necrosis or autophagy, only become visible when apoptosis is inhibited. Treatment of the cells with the various extracts caused limited morphological changes, and it was mainly the Chinese kernel extracts that caused changes that could be associated with apoptosis. Changing of intracellular ATP levels can affect the form of cell death. High ATP levels lead to apoptosis, while a low ATP level leads to necrosis confirming that an intracellular ATP depletion switches the energy-dependent apoptotic cell death to necrosis. As cyanide acts through the inhibition of cytochrome-c oxidase in the respiratory electron transport chain of the mitochondria, impairing both oxidative metabolism and the associated process of oxidative phosphorylation causing death through energy deprivation, it is possible that ATP levels decreased in HT-29 cells influencing the type of cell death observed after the kernel treatment switching the energy-dependent apoptotic cell death to necrosis.

Regulation of the cell cycle is important in the growth and development of cancer. Cell cycle progression revealed a significant S-phase peak in the cells treated with SAK and SPK. The intra-S phase checkpoint activated by genotoxic insults causes only temporary, reversible delay in cell cycle progression, mainly by inhibition of new replicons initiation and thereby slowing down DNA replication, but not permanently arresting DNA replication [30].

Bladder cancer cells [﻿27﻿] exposed to amygdalin only, showed a significant delay in cell cycle progression and a G_0_/G_1_ arrest whereas the HT-29 cells showed the induction of an intra-S phase block which is shown by an increase in the number of cells in the S phase and a decrease in the number of cells in the G_1_ and G_2_ phases. The accumulation of cells in the S-phase started at 24 and continued throughout the different time and doses indicating that the South African kernels affect the cell cycle progression possibly by activating the intra-S-phase checkpoint. Activation of the intra-S-phase block slows down the processes of DNA synthesis allowing time for DNA repair thus preventing genomic instability. A long term intra-S-phase block (48 h) could limit the amount of sister chromatids and therefore reduce available templates for efficient repair by homologous recombination [30, 31]. Complete inhibition of cell cycle regulating enzymes and prolonged intra-S phase arrest may cause regaining of replication competence of already fired origins, making the recovery process prone to over-replication of at least parts of the genome. It is therefore possible that the insult caused by SAK and SPK treatments could act by inducing this block that could later progress to cell death [﻿31﻿].

The organic extractions of the Chinese apricot kernels showed an increase in the number of cells in the G_0_/G_1_ and no relative increase in cell numbers in the S-phase as compared to the Chinese peach kernels which showed a slight increase in the number of cells in the S-phase.

The inhibitory effect of the Chinese kernels lasted for 48 h only. Therefore, repetitive treatment of the cells every 24 h or 48 h would greatly reduce and maintain the reduction of colon cancer growth. It can therefore be deduced that the inhibition of proliferative activity was not due to a permanent cytotoxic effect of the kernels, but rather a modulation of cell cycle progression and the induction of a temporary S-phase block. Mitosis is therefore influenced in a different manner depending on the type of kernel extraction, dose and time of exposure. Future studies will include determination and quantification of the specific type of cell death induced by the different extractions. The presence of S-phase block will also be verified by studying changes in cyclin A levels.

## Conclusion

The growth inhibition effect of the CAK and CPK extracts may be due to increased necrosis and possibly autophagy as an alternative to decreased proliferation in the cells. This possibility should be evaluated in further studies. Low ATP levels, caused by the presence of amygdalin in the kernels, can also switch to pycnosis or necrosis. Willis and Rhind 2009 [32] suggest that the intra S-phase checkpoint may be more concerned with tolerating and accommodating damage during replication rather than repairing it, thus the HT-29 cells most probably proceed to necrosis in the presence of outspoken DNA damage by specially the high concentrations CAK/CPK (24 h and 48 h) and SAK after 72 h.

Cancer cells are thought to have more β-glucosidase and less of the liver enzyme rhodanese which converts cyanide to relatively harmless compound thiocyanate. Thus the cancer cells are more susceptible to the effect of amygdalin than the healthy cells. Daily intake due to the narrow therapeutic index and reduced risk of toxicity based on the form of administration and preparation may make these kernels a viable chemopreventive agent.

## Abbreviations

CAK: Chinese apricot kernel
CPK: Chinese peach kernel
CRC: Colorectal cancer
FA: Fatty acids
GAE: Gallic acid equivalent
Hydro: Hydrophilic
IC_50_: 50% inhibitory concentration
Lipo: Lipophilic
SAK: South African apricot kernel
SD: Standard deviation
SPK: South African peach kernel
Tot: Total
